# Laryngeal Crepitus as a Sign of Pulmonary Tuberculosis

**Published:** 1904-05-14

**Authors:** 


### LARYNGEAL CREPITUS AS A SIGN OF PULMONARY TUBERCULOSIS.

Under the term of "laryngeal crepitus" Dr. Remouchamps[1] describes a physical sign which he believes to have considerable diagnostic value in early cases of pulmonary tuberculosis, having assured himself of its presence in upwards of a hundred phthisical patients. The method of observing it is as follows:—The patient stands face to face with the physician, and is directed to open his mouth, and placing his right hand on the patient's left shoulder, and resting his left thumb on the patient's chin, the physician brings his left ear to within 2 or 3 inches of the patient's mouth. He will then be able to perceive, in cases of pulmonary tuberculosis, a fine crepitation, which Dr. Remouchamps terms "laryngeal" because its maximum intensity appears to be in the larynx. The sound resembles that of a fine pen slowly scratching on paper. It is audible both in inspiration and expiration, but is more perceptible during the latter. The crepitation is present and audible, the author declares, throughout the whole course of phthisis, increasing, diminishing, or disappearing according as the illness progresses, or is recovered from. In conclusion, Dr. Remouchamps expresses the conviction that persistent "laryngeal crepitus" is pathognomonic of pulmonary tuberculosis from its very onset, also that, in a case where the diagnosis remains doubtful, the persistent absence of this sign justifies the exclusion of phthisis.

[1] Med. Press, April 20.

